# Significance of intra-fractional motion for pancreatic patients treated with charged particles

**DOI:** 10.1186/s13014-018-1060-8

**Published:** 2018-06-25

**Authors:** Vania Batista, Daniel Richter, Naved Chaudhri, Patrick Naumann, Klaus Herfarth, Oliver Jäkel

**Affiliations:** 1 Heidelberg University Hospital, Heidelberg, Germany; 2 Erlangen University Hospital, Erlangen, Germany; 3 Heidelberg Ion-Beam Therapy Center, Heidelberg, Germany; 4 GSI Helmholtz Centre for Heavy Ion Research, Darmstadt, Germany; 5 German Cancer Research Center, Div. Medical Physics in Radiation Oncology, Heidelberg, Germany; 6 Heidelberg Institute for Radiation Oncology (HIRO), National Center for Radiation Research in Oncology (NCRO), 69120 Heidelberg, Germany; 7 RadioOnkologie und Strahlentherapie, Universitätsklinikum Heidelberg, Im Neuenheimer Feld 400, 69120 Heidelberg, Germany

## Abstract

**Background:**

Uncertainties associated with the delivery of treatment to moving organs might compromise the accuracy of treatment. This study explores the impact of intra-fractional anatomical changes in pancreatic patients treated with charged particles delivered using a scanning beam. The aim of this paper is to define the potential source of uncertainties, quantify their effect, and to define clinically feasible strategies to reduce them.

**Methods:**

The study included 14 patients treated at our facility with charged particles (protons or 12C) using intensity modulated particle therapy (IMPT). Treatment plans were optimized using the Treatment Planning System (TPS) Syngo® RT Planning. The pre-treatment dose distribution under motion (4D) was simulated using the TPS TRiP4D and the dose delivered for some of the treatment fractions was reconstructed. The volume receiving at least 95% of the prescribed dose (V95CTV) and the target dose homogeneity were evaluated. The results from the 4D dose calculations were compared with dose distributions in the static case and its variation correlated with the internal motion amplitude and plan modulation, through the Pearson correlation coefficient, as well the significant *p*-value. The concept of the modulation index (MI) was introduced to assess the degree of modulation of IMPT plans, through the quantification of intensity gradients between neighboring pencil beams.

**Results:**

The induced breathing motion together with dynamic beam delivery results in an interplay effect, which affects the homogeneity and target coverage of the dose distribution. This effect is stronger (∆V_95CTV_ > 10%) for patients with tumor motion amplitude above 5 mm and a highly modulated dose distribution between and within fields. The MI combined with the internal motion amplitude is shown to correlate with the target dose degradation and a lack of plan robustness against range and positioning uncertainties.

**Conclusions:**

Under internal motion the use of inhomogeneous plans results in a decrease in the dose homogeneity and target coverage of dose distributions in comparison to the static case. Plan robustness can be improved by using multiple beams and avoiding beam entrance directions susceptible to density changes. 4D dose calculations support the selection of the most suitable plan for the specific patient’s anatomy.

**Electronic supplementary material:**

The online version of this article (10.1186/s13014-018-1060-8) contains supplementary material, which is available to authorized users.

## Background

Treating pancreatic cancer is still an oncological challenge, it being one of the deadliest cancers worldwide [1, 2]. The use of photon irradiation is limited due to the close proximity of the pancreas to the duodenum. Radiotherapy with charged particles has been considered a promising approach to improving patients’ overall survival rates [3, 4]. This is because the sharp dose gradient may allow for dose escalation. Nevertheless, uncertainties can compromise the accuracy of this treatment to a greater extent than is the case for conventional irradiation. These uncertainties originate from anatomical changes between treatment sessions (inter-fractional changes), the positioning of the patient, internal motion of the patient’s organs during the delivery of treatment (intra-fractional), and beam application uncertainties (range, position and width of pencil beams). The considerable sensitivity of the ion range to density changes in the beam-path reduces the tumor coverage, increases the dose inhomogeneity and may cause an overdose in normal tissues.

Anatomical changes during the course of the treatment, as well as tumor volume changes, intestine and stomach filling and loss of adipose tissue, have been discussed in a recent publication [5].This study, however, will address the impact of intra-fractional changes.

Intra-fractional anatomical variations, i.e. the induced breathing motion, together with dynamic beam delivery, has been shown to affect the dose distribution in terms of homogeneity and target coverage [6]. This so-called interplay effect must be quantified for each pathology and facility-specific configuration of the beam delivery system.

The integration of the motion information in the treatment planning can be accomplished through a time-resolved (4D) treatment planning system (TPS). The 4DTPS simulates the temporal interference between the beam and the target motion, as given by an external surrogate signal. Information about the patient is taken from a 4DCT, while the beam delivery sequence (BDS), i.e. number of particles per spot, intensity level and beam pauses, is obtained from the accelerator control system. When the BDS and breathing signal are measured during treatment, a time-resolved dose calculation, known as *4D Dose Reconstruction* (4DDRec), may be performed. When a simulated BDS is used, the dose determination will be referred to as *4D Dose Simulation* (4DDSim) [7].

When it comes to the challenging anatomical location of pancreatic tumors, surrounded as they are by multiple organs-at-risk (OARs), Intensity Modulated Particle Therapy (IMPT) offers the benefit of allowing the dose gradients to be increased between the OARs and the tumor. However, IMPT has greater potential to facilitate an increase in the effect of range and set-up uncertainties than the Single Field Uniform Dose (SFUD) plans [8]. In the context of photon therapy, the concept of a modulation index was suggested as a way of quantifying the modulation of the plan fluency [9]. In this study, this parameter was adapted to scanned particle beams in order to assess the robustness of IMPT plans and correlate this with the interplay strength.

## Methods

### Patient cohort, imaging and immobilization technique

The breathing signals and beam delivery sequence of fourteen pancreatic patients was monitored during irradiation. The free-breathing planning CTs (CT_plan_) and 4DCTs were acquired in the Somaton Sensation Open scanner (Siemens, Erlangen, Germany), which performs a relative phase-based reconstruction on the basis of the surrogate signal of the motion-monitoring system AZ-733 V Respiratory Gating System (Anzai Medical Co.,Ltd., Japan), herewith referred to as “Anzai”. The 4DCT images were sorted in eight standard motion states, using the breathing phases (0%Ex, 40%Ex, 70%Ex, 100%Ex, 75%In, 50%In, 25%In and 20%In), where *In* corresponds to the inspiration and *Ex* to the expiration process. The state 0%Ex is the end-exhale and 100%Ex is the end-inhale state. A sample of the breathing signal, with the length of a typical treatment, was acquired for the majority of the patients during the CT session. A description of the set of patients is available in Table 1.

**Table 1:** Description of the set of patients, containing the information of the total dose prescription (T.dose), and per fraction (F. dose), particle used (protons or carbon ions), existence of pre-treatment breathing signal (y- yes, n- no), number of treatment fractions with recorded monitoring (Fx.monit). The median vector field length for the most extreme breathing state to end-expiration (0%Ex) CT is for each patient 4DCT inside the ITV calculated (Max.MedianVFL). The adopted beam configuration (B.Config) follows the naming of the Fig. 1

Patient	T.Dose Gy (RBE)	Fx.dose Gy (RBE)	Particle	B.Config.	Pre-tt breath. signal	Fx.Monitor.	Max. MedianVFL (mm)
H1	45 + 9	1.8	p	B	Y	2	8.7
H2	45 + 9	1.8	p	B	Y	1	6.9
H3	45 + 9	1.8	p	C	Y	1	4.6
H4	45 + 9	1.8	p	B	Y	3	3.1
H5	45 + 9	1.8	p	B	Y	4	3.3
H6	45 + 9	1.8	p	B	N	3	4.5
H7	54	2	p	B	N	1	4.6
H8	45 + 9	1.8	p	B	Y	1	4.1
H9	45 + 9	1.8	p	B	Y	1	4.7
H11	45 + 9	1.8	p	B	Y	3	5.0
H12	45 + 9	1.8	p	A	Y	6	12.7
H13	48	4	^12^C	B	Y	2	5.0
H14	48	4	^12^C	B	Y	1	3.1
H15	45 + 9	1.8	p	B	Y	1	2.2

Patients were immobilized, lying in a prone position, using a vacuum mattress. This positioning resulted from the need to use irradiation with posterior beams, in order to reduce the inter-fractional anatomy variations in the delivered dose [5], and a limitation of our beam delivery system at the time (no accurate delivery of beams coming through the treatment table and indexing support). As a consequence of this prone immobilization, no abdominal compression was applied and the patients were imaged and irradiated under free-breathing.

The patient position was verified in-room by a 2D-3D bony anatomy image registration between the orthogonal X-ray taken at the isocenter and the DRRs calculated from the planning CT. This enabled the translational and rotational shifts to be determined meaning that the patient could be accurately positioned on the treatment couch.

### Treatment plan

Treatment planning was performed using the TPS Syngo® RT Planning, which uses the LEM model for effective dose calculation of the carbon ions and a fixed RBE factor of 1.1 for protons. In general, the plans were optimized using IMPT for an initial dose of 45 Gy (RBE) - 54 Gy (RBE) with an additional boost of 9 Gy (RBE) for some cases, as specified in Table 1.

A scanning raster spacing of 3 × 3 mm in the lateral direction, and an iso-energy slice spacing of 3 mm water-equivalent was used for both the proton and carbon plans. The initial optimization parameter for the pencil beam focus was 8 mm FWHM for the proton beams (range between 8 and 30 mm depending on energy). For the carbon ion beams, however, a maximum width of 10 mm FWHM was selected (range between 6 and 10 mm). These parameters were chosen in view of the results from a previous study [10], in which the interplay effects were minimized for an enlarged FWHM of the pencil beam.

**Fig. 1:**
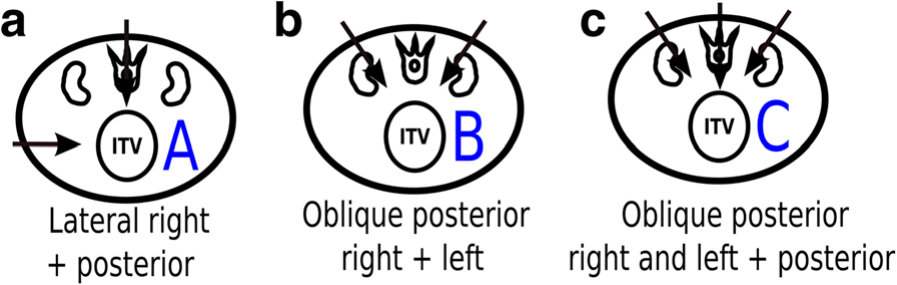
Beam configurations (**A** , **B** , **C**) adopted for these patients, in which the patients were prone positioned

The selected beam configuration for each patient was consequence of: (i) the superior inter-fractional robustness of ion-beams posterior to the patient (according to [5]); and (ii) the need to sparing the OARs (spinal cord and kidneys) from unwanted doses. It was therefore treated twelve of the fourteen patients with two posterior oblique fields. The remaining two patients were treated with a different geometry due to OARs constraints. Treatment was nonetheless considered robust from the inter-fractional point of view. The beam arrangements used are illustrated in Fig. 1.

In all cases, plans were optimized to the PTV in order to deliver the prescribed dose (D_presc_) to the CTV while keeping the OARs doses below the dosimetric constraints of the spinal cord, kidneys and intestines. Due to the short distance between the tumor bed and the intestine, the prescribed dose was not achieved for all the patients over the entire CTV.

The PTV was assigned as an ITV expansion, by 7 mm in beam direction and 5 mm laterally, while the ITV corresponds to the union of the CTV in each of the 4DCT phases.

### Image registration

The 4DCTs were rigidly registered using the bony anatomy of the CT_plan_. Deformable image registration (DIR) was performed between the CT_plan_ and the reference 4DCT state, CT_0Ex_, with the aim of contour propagation using the vector field obtained. Moreover, each of the 4DCT states was registered against the CT_0Ex_ with the objective of deriving motion information during the calculation of the time resolved dose distribution. The DIR was performed with Plastimatch, using two successive registrations with a B-Spline algorithm [11]. The quality of the 4DCT DIR was assessed using the platform 3D Slicer [12], in particular using the Registration Quality Module [13], which was developed by external contributors as a set of tools that can be incorporated into 3D Slicer. The evaluation was performed through visual inspection and numerical quantification, such as the determinant of the Jacobian matrix (JD) of the vector field, inverse consistency error (ICE) and mean absolute difference.

### Breathing signal and irradiation sequence

A pre-treatment acquisition of the breathing signal was performed for twelve of the patients during the CT_plan_ acquisition session, as indicated in Table 1. For the other two patients, the signal wasn’t acquired during the CT session. As such, a standard Lujan motion with a patient representative period of 3 s was considered [14].

The beam delivery structure was simulated using a tool developed in-house, makeLmdout-MH [7, 15], based on the synchrotron base data. The base data was obtained from irradiated plans and considers the acceleration times, energy dependence and random intensity fluctuations. The output of this tool is the random simulation of the accelerator timing and intensity patterns for the given plan.

The inputs for the tool are the optimized treatment plan, the breathing signal and the accelerator spill information. The spill was characterized by the maximal extraction time of 5.0 s, pause length and pause length at the end-of-plan of 4.2 s (i.e. the time set to start a new spill within the same IES, and the beam pause when a IES is finished and the beam goes to the next IES, respectively).

As output, a simulated BDS is obtained, which will be given as input for the 4D dose calculation. In order to describe the spectrum of possible irradiation scenarios [16, 17], which results in different interplay patterns, a temporal shift to the starting phase of the surrogate signal was applied, i.e. a temporal delay between the starting of the breathing signal. This will correspond to the irradiation of a different raster point in a defined breathing phase. These shifts were spaced 500 ms in a total of five different starting points of the irradiation for the pre-treatment breathing signal and are given as input for the 4DDSim.

During the patient irradiation, the Anzai system was used to monitor motion. This system was connected to a data acquisition system, known as the EtherCat system, which correlated the breathing signal and the beam delivery temporal sequence of the accelerator in time. In order to improve the acquisition statistics, the different intensity rate from the proton and carbon beams was considered and the sampling time was defined as 0.15 msec and 0.25 msec for protons and carbon ions respectively. The calculation of 4DDRec was therefore performed on the basis of the measured data (breathing and irradiation sequence) during the irradiation of the individual treatment fractions. The number of available fractions with monitoring data is listed in Table 1.

### Time resolved forward calculation of the dose distribution

The calculation of 4DDSim and 4DDReco was performed using TRiP4D [17, 18]. The forward calculation was based on the treatment plan information (raster points, energies and beam focus), breathing signal and the accelerator temporal pattern, either simulated or measured, respectively. In addition, the vector fields obtained for the DIR between each of the 4DCT states and the reference state (CT_0Ex_) were given as input.

For both particles types, the forward dose calculation followed the same parameters as in the Syngo® RT TPS, differing for the proton plans only, where the physical or absorbed dose was computed in TRiP4D. However, in order to render negligible the effect of differences between the beam models, the dose distribution was also calculated in the static case, i.e. for the CT_plan_, and this dose distribution was taken as reference for the comparison.

### Evaluation methods

The internal tumor motion of each patient was quantified using the vector field obtained from the DIR between the CT_0Ex_ and each of 4DCT states, and in particular by measuring the median vector field length (VFL) inside the ITV_0Ex_. The maximum of these values was used as a quantification of the intra-fractional tumor motion, generally corresponding to the CT_100Ex_.

The dose distributions, namely the static, the 4DDSim, and 4DDReco, were evaluated by taking as metric the volume receiving at least 95% of the prescribed dose (V_95CTV_) and the target dose homogeneity (H_CTV_ = D_5_-D_95_).

Note that the 4DDSim corresponds to a set of dose distributions, as representative of different interplay patterns, resulting in the need to display the results as mean and standard deviations and the DVHs as band-DVHs.

In order to simplify the analysis, only the initial plan was considered in the evaluation and the dose distribution for the boost plan was ignored.

In order to evaluate the impact of the dose modulation on the plan robustness to intra-fractional changes and interplay events, the normalized variation of the number of particles per irradiation field was evaluated (${\overline{\sigma np}}_{field}$). This parameter is given by eq. (1). In (1) mean_np,field_ is the mean number of particles (np) for the respective field and σ_np_ is the respective root-mean-square of the mean of the squared differences between the number of particles at each IES (i_ies) and raster point (i_rp) in the total number iso-energy slices (nIES) and all the raster points in each IES (nrp). The parameter nRP is the total number of raster points for the evaluated field.

1 $$ {\overline{\sigma np}}_{field}=\frac{\sqrt{\frac{1}{nRP}{\sum}_1^{i\_ ies= nIES}{\sum}_1^{i_{rp}= nrp}{\left({np}_{i\_ ies,i\_ rp}-{mean}_{np}\right)}^2}}{mean_{np, field}}=\frac{\sigma_{np}}{mean_{np, field}} $$

In addition, to account for variations between adjacent raster points, the concept of Modulation Index (MI) was applied (eq. 2a). The MIs were calculated from the treatment plan information of each field (MI_field_), given by the raster points (rp) intensity and location.

This index accounts with the changes in adjacent raster points through the calculation of a function F (eq. 2b). Here, for each raster point, the magnitude of the difference between its intensity and the intensity of neighboring raster points is calculated through *∆* = |*I*_*rp*_ − *I*_*rp* − 1_|.

2a $$ {MI}_{field}={\int}_{\mathrm{i} es=1}^{ies= nIES}F{(IES)}_{\delta } $$

where

2b $$ F{(IES)}_{\delta }=\frac{N_{\Delta  >\delta }}{{\left( nrp-1\right)}_{IES}} $$

Secondly, the number of raster points (nrp) in each IES, whose ∆ is above a factor, δ, of the variation of its IES is counted. This parameter is called N.

In brief, the function F quantifies the modulation of a plan by the measure of changes in adjacent raster points that exceed a certain fraction of the variation in each IES. Hence, the area of this spectrum of deviations, namely the area below the F function, gives the degree of modulation i.e. MI.

The value of δ was selected as 1.2, in an iterative process in a way to be sensitive to variations of the number of particles between adjacent raster points. For this purpose, the value of δ was varied, and the resulting function F was compared with the dose distribution per beam. For clinically homogeneous plans, therefore the function F has a small value, while it becomes gradually larger for regions with larger dose gradients.

As both parameters are applied per field, a weighted mean per plan for the different fields was used, giving the parameters ${\overline{\sigma np}}_{plan}$ and MI_plan_. The weighting was approximated in view of the number of particles per beam.

To assess the correlation between the plan parameters (V_95CTV_, H_CTV_, MI_plan_, ${\overline{\sigma np}}_{plan}$) and the motion vector magnitude, a multi-pairwise analysis was performed. For this purpose, the Pearson linear correlation coefficient (r) for each pair of variable and respective significance (*p*-value) were calculated. Correlations with a p-value < 0.05 were considered significant. The entire statistical evaluation was performed using R libraries [19].

## Results

### Internal motion

**Fig. 2:**
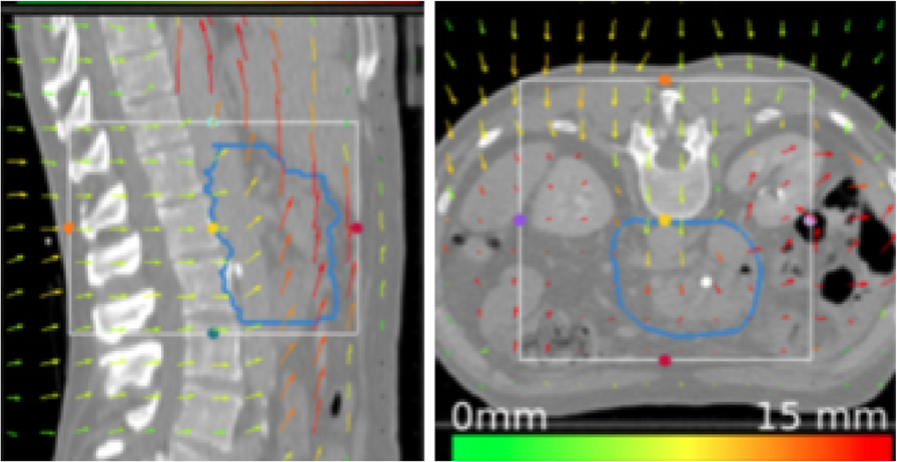
Vector field representation obtained from the deformable image registration between the end- and full-exhale state for the patient H1. The vector direction represents the deformation of voxel between CTs, while the color indicate the magnitude of the deformation

The median vector field length inside the ITV is shown in Table 1. The median of the vector field for this set of patients was (5.2 ± 2.7) mm, ranging from 2.2 to 12.7 mm. The main component of the motion was detected in the cranio-caudal direction, followed by the anterior-posterior direction. Figure 2 shows the vector field for the patient H1.

### Simulated time resolved dose distribution

In order to eliminate differences in dose calculation between TRiP4D and Syngo® RT the shown evaluation of the 4D dose distributions is the comparison to the static dose distribution also calculated with TRiP4D. Note that the results for the 4DDSim and 4DDReco correspond to the propagated CTV (CTV_0Ex_) contour from the CT_plan_ to the reference state CT_0Ex_.

**Fig. 3:**
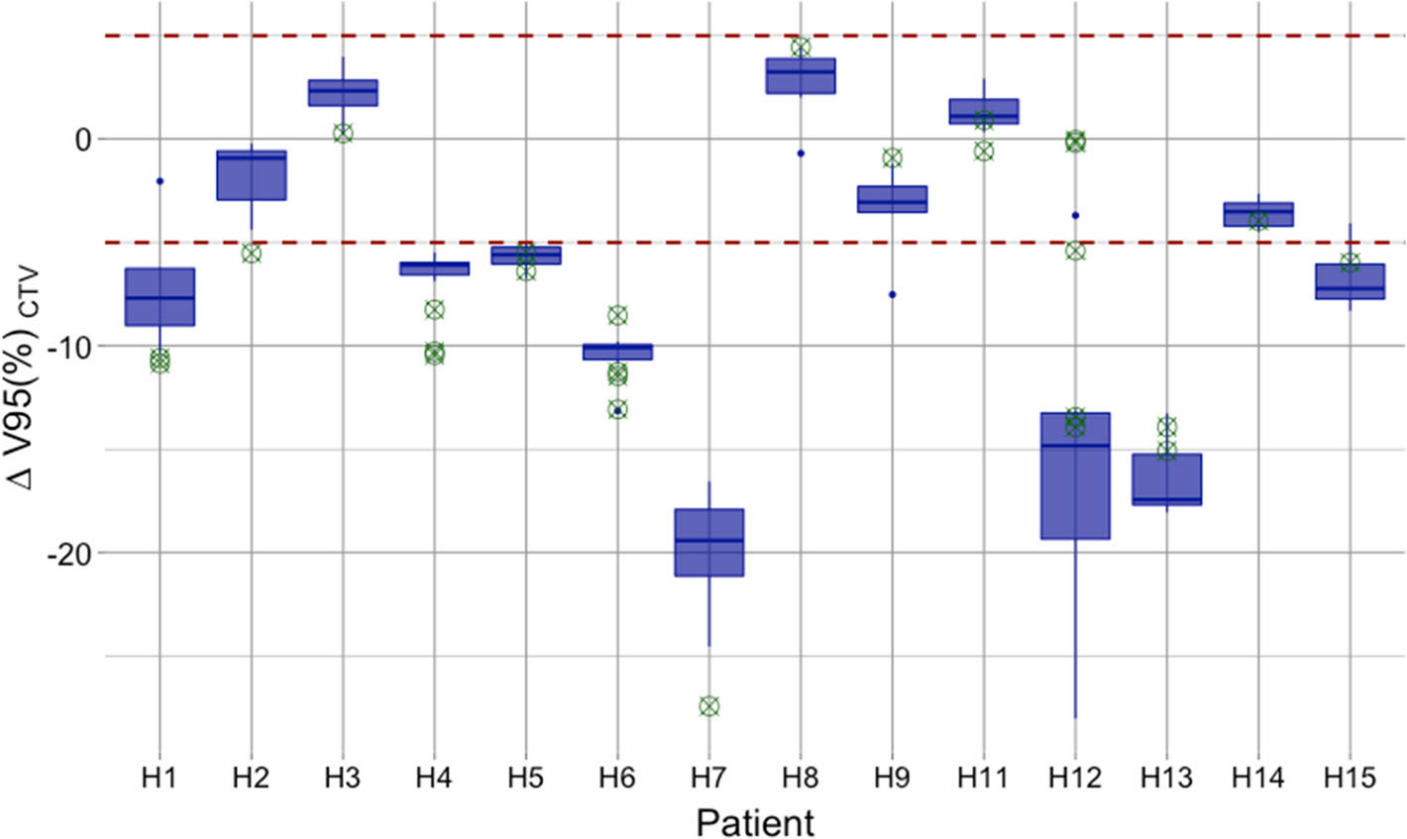
Differences of the V_95CTV_ for all the patients relative to the static dose distribution value. Each boxplot corresponds to the five simulated interplay patterns from the 4DDSim, while the green crosses are the results from each of the treatment fraction where a 4DDReco was performed. The red dashed lines define the 5% of variation relative to the static dose distribution and are here used to help in the detection of the patients with major deviations

Figure 3 illustrates the overall results. At first glance, these results seem to show that a large number of plans were strongly affected by beam interplay and displacements. In the simulated cases, the variation of theV_95CTV_ reached values of up to − 28.0% with a mean of (− 7.6 ± 7.6)%. The H_CTV_ was also impaired, increasing from (15.9 ± 7.5)% in the static case to (27.8 ± 8.5)% under motion.

**Fig. 4:**
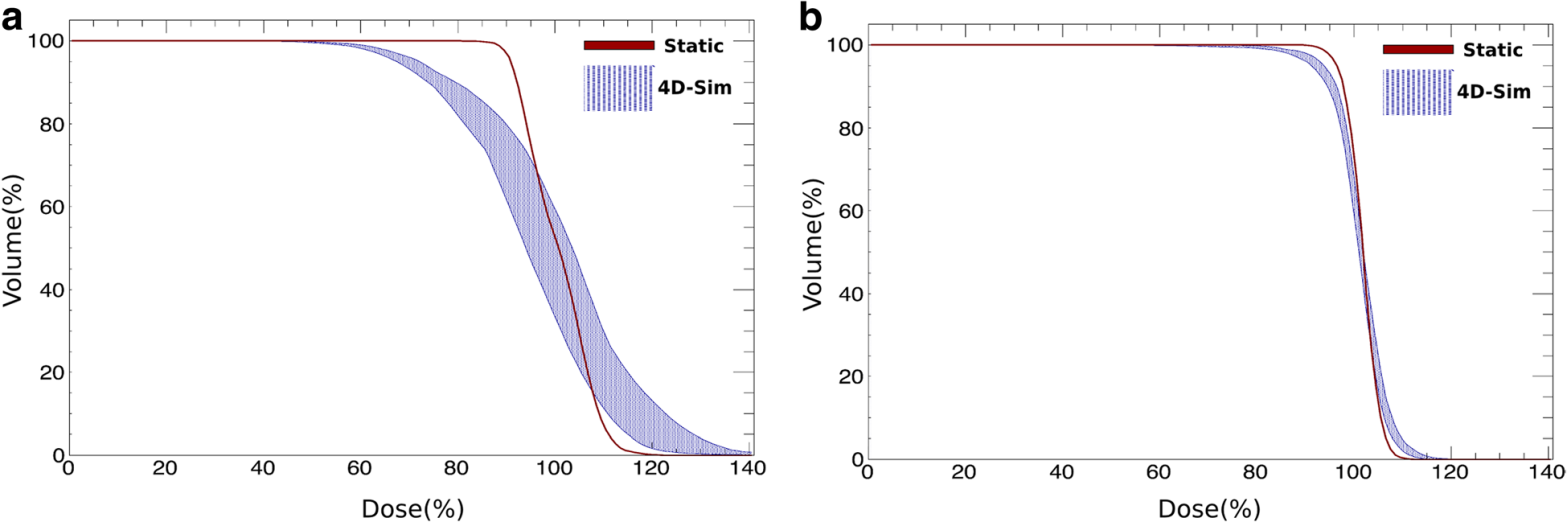
DVH of the patient H12 (**a**) and H15 (**b**) for the CTV_0Ex_ in the static case (red line) and for the set of 4DDSim as the blue band

Guiding the interpretation of these results, Fig. 4 shows the DVH for the CTV of the reference dose distribution (i.e. static) and of the set of 4D simulations, for the two patients with the largest and smallest internal motion. Patient H12, due to a large internal motion (> 10 mm), shows a broad DVH and a mean reduction of the V_95CTV_ of (− 15.8 ± 8.1)%. In contrast, patient H15, with a mean tumor motion below 3 mm, shows a reduction in the V_95CTV_ of (− 6.7 ± 1.6)%, not being expected high dose variations between different treatment sessions.

Our analysis shows that the dose degradation is affected by the internal motion amplitude, with a strong correlation between the motion amplitude within the tumor and the standard deviation of the V_95CTV_ variations relative to the static case (*r* = 0.86, *p*-value < 0.05). However, we also see a non-significant correlation with the mean V_95CTV_ variations relative to the static case (p-value > 0.05). The homogeneity dose, H_CTV_, was seen to be more sensitive to motion, with the mean and standard deviation differences strongly correlated (*r* = 0.61 and 0.77, respectively, p-value < 0.05).

**Fig. 5:**
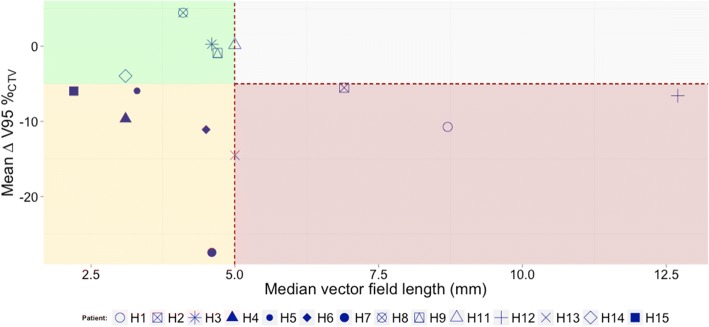
Mean difference of the V_95CTV_ between the static and the 4DDSim versus the median vector field length inside the ITV. Red region corresponds to large motion and consequently higher dose degradation, while green are patients with a robust dose distribution against intra-fractional motion. The yellow region corresponds to patients where the motion amplitude is small (< 5 mm) but a reduction in the V_95CTV_ is demonstrated. A region without cases was found, grey area, which corresponds to any patients with large motion and small V_95CTV_ variations

The variation of the V_95CTV_ is represented against the internal motion amplitude in Fig. 5. The patients were categorized in three groups: red (> 5 mm motion and > 5% of CTV dose degradation), yellow (large motion, i.e. > 5 mm), and green (reduced impact on the dose distribution and motion below 5 mm). The definition of these limits represents the clinical practice at our facility.

This comparison suggests that as expected, patients belonging to the red group show a reduction in the target coverage (reduced mean variation of the V_95CTV_ relative to the planned dose distribution) throughout the entire course of treatment. Other patients however, such as H7, do not support this hypothesis. In fact, we observed that five patients for whom the motion amplitude was below 5 mm the target suffered strong dose degradation (yellow region). Another conclusion was that no patient with a large internal motion (> 5mm) showed small dose distribution degradation, i.e., no patients were observed in the grey region in Fig. 5). This justifies the need to monitor the motion amplitude for pancreatic patients throughout treatment, applying an appropriate strategy to reduce its impact (e.g. gating, robust optimization, rescanning, etc.).

### Reconstructed time resolved dose distribution

The evaluation of the 4DDReco is also shown in Fig. 3, where each green cross represents one treatment fraction, overlaid with the static and 4DDSim results. This figure indicates that the 4DDSim resulted in a good approximation of the robustness of the plan for some treatment fractions, while for others it can be used as an indicator of the probability of seeing a reduction of the CTV dose, either by the mean or width of the boxplot of a set of simulations. The mean V_95CTV_ obtained from the 4DDSim strongly correlates with the mean V_95CTV_ from the set of 4DDReco (*r* = 0.87, *p*-value < 0.05).

**Fig. 6:**
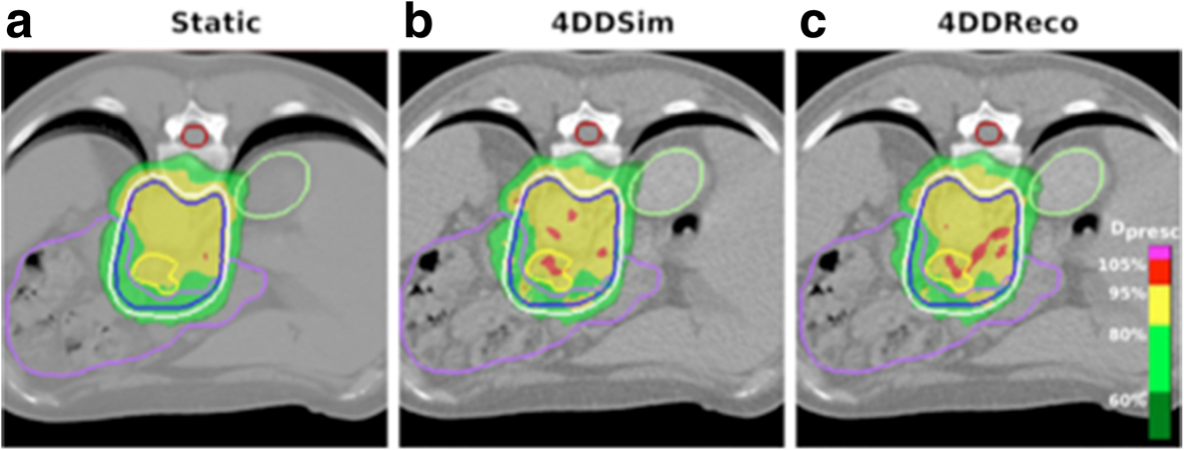
Dose distribution in the transversal CT view for the patient H3 in the static (**a**), one of the simulated cases (**b**) and in the reconstructed fraction (**c**). In yellow, blue and white, the GTV, CTV and ITV are displayed, respectively. The dose distribution was tailored in order to keep the bowel doses (in purple) below the dosimetric constraints. In (**a**) is shown the planning CT, while in (**b**) and (**c**) is the CT_0Ex_

Figure 6 shows an example (patient H3) of the dose distribution at one axial slice in the static, 4DDSim and 4DDReco situation, in which similar results to the 4DDSim and 4DDReco are seen, i.e. increase of the dose inhomogeneities and reduction of the target coverage dose. Nevertheless, other patients (such as H7 and H12) exhibit a 4DDReco for a specific fraction outside the predicted set of 4DDSim.

In general, patients with minor internal motion tend to have more similar 4DDSim solutions, i.e. a small interplay effect and therefore a small box width in Fig. 3. However, the number of calculated simulations has limited value for the description of all possible interplay patterns over and above those detected during the 4DDReco. It would be necessary to carry out further simulations in order to cover a larger range of solutions. Nonetheless, the 4DDSim results presented here, do indicate whether a plan is or is not robust (high correlation found between 4DDSim and 4DDReco).

From the visual inspection of the example dose distributions, patient H3 shown in Fig. 6, one can observe that the static plans were highly modulated for this patient. This effect was also observed for other patients. This was associated with the dose optimization constraints of the OARs (mainly bowel) and target coverage, which result in sharp dose gradients between the tumor and the bowel contour. Hence, another studied conjecture was the influence of the plan modulation on the plan robustness to the breathing motion.

### Impact of dose modulation

The normalized standard deviation of the number of particles (${\overline{\sigma np}}_{plan}$), the modulation index and the variation of the V_95CTV_ and H_CTV_ for all the patients and plans are presented in Table 2. The significant linear correlations between parameters are also seen here. The values per patient are available in the Additional file 1: Table S1.

**Table 2:** Statistical analysis of the variation of the magnitude of the internal motion vector within the tumor, the variation of the target coverage (indicated by the V95_CTV_ parameter), the dose homogeneity (H_CTV_), average of the variation of the number of particles per IES ($\overline{\overline{\upsigma \mathrm{np}}\ }$) and Modulation Index (MI_plan_). The values presented correspond to the mean, standard deviation (std.) and the two extreme cases (minimum and maximum) for the set of plans and patients. Each of these parameter was between each other correlated, the Pearson correlation coefficient (r) and the significance p-value are presented. Correlations with *p*-values below 0.05 were considered not significant (n.a.)

Parameter	Mean ± std.	Min.	Max.	Correlation Parameter	r (p-value)
Internal Motion vector magnitude (mm)	5.2 ± 2.7	2.2 (H15)	12.7 (H12)	Std. ∆V_95_	0.86 (< 0.05)
				Std. ∆H_CTV_	0.77 (< 0.05)
∆V_95_4DDSim + 4DDReco (%)	−6.9 ± 7.0	1.2 ± 1.0 (H11)	−20.9 ± 3.9 (H7)	$\overline{\upsigma \mathrm{np}}$	n.a. (> 0.05)
				MI	n.a. (> 0.05)
				MI*motion	0.76 (< 0.05)
∆H_CTV_4DDSim + 4DDReco (%)	11.7 ± 8.9	0.6 ± 1.0 (H11)	30.0 ± 5.9 (H12)	$\overline{\upsigma \mathrm{np}}$	n.a. (> 0.05)
				MI	n.a (> 0.05)
				MI*motion	0.75 (< 0.05)
$\overline{\upsigma \mathrm{np}}$	1.7 ± 0.4	1.1 (H13)	2.6 (H8)	–	–
MI	11.2 ± 6.2	3.0 (H14)	22.5 (H11)	–	–

**Fig. 7:**
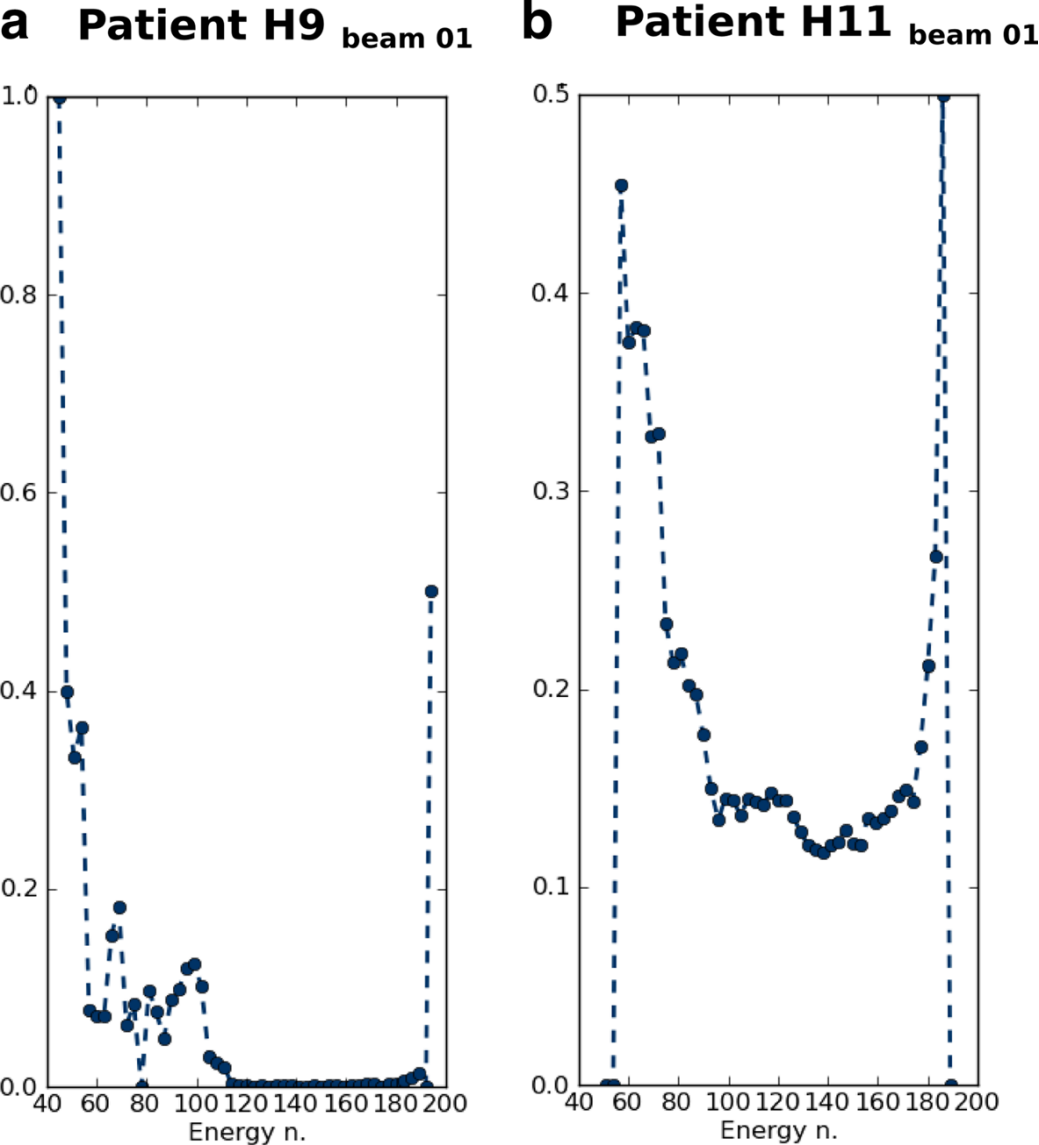
Distribution along the iso energy slice (IES) of the modulation function of the number of particles for one of the treatment fields for the patient H9 (**a**) and H11 (**b**)

An example of these MI_field_ variation patients, namely H9 and H11, are presented in Fig. 7. These patients’ plans were selected because although both of them exhibit the same amount of tumor motion (median VFL inside the ITV), their 4D dose distribution varies significantly. In Fig. 7, the function of the modulation, F, in which the MI_field_ corresponds to the area below the curve, is represented as a function of the IES for these cases. In both cases, it was observed that the Syngo® RT optimizer tended to have a strong modulation at tumor borders, as a result of an optimization resembling distal edge tracking. This effect is stronger; that is, more IESs show a higher F value, when the tumor is in the proximity of OARs, as in H11. Where this is not the case, the shape of the function is similar to the one seen for patient H9, where the first and last IES show a higher F value and the values in-between F are close to zero. Syngo® RT prioritizes the OARs constraints against the tumor irradiation, resulting in an increase of the MI_field_ when more constraints for the OARs are defined. Moreover, Syngo® RT uses a Broyden-Fletcher-Goldfarb-Shanno (BFGS) algorithm to solve the optimization problem. The solutions found by the BFGS algorithm, however, do not include regularization of the number of particles between neighboring raster points (regularization means a smoothing of the distribution of particle numbers in the target volume). This allows a greater difference between the particle numbers in neighboring raster points. For the set of patients the MI_plan_ using the TPS Syngo® RT was 11.2 ± 6.2. In comparison, the common values obtained for the other set of patients with the TPS TRiP4D and different constraints were of 1.8 ± 2.6. This indicates that different optimizers and different optimization constraints might result in contrasting modulation levels. Having said this, this comparison is beyond the scope of this study, as only a certified TPS is used for clinical treatment optimization.

The statistical evaluation of the data showed that ${\overline{\sigma np}}_{plan}$ and MI_plan_ do not exhibit a significant linear correlation with the variation of the H_CTV_ or V_95CTV_ (*p*-value > 0.05).

The difference between these two concepts ($\overline{\sigma np}$ and MI) is that $\overline{\sigma np}$ ignores the location of the raster points and may not be representative of intensity differences between neighboring points and the plan modulation. MI does not, however, include the energy information and the use of multiple beams may diminish its significance. Nevertheless, when this parameter is weighted by the internal motion magnitude, it becomes highly correlated with variations in the target coverage and inhomogeneity, *r* = 0.76 (p-value 0.002) and *r* = 0.75 (p-value 0.001), respectively for the standard deviation of the V_95CTV_ and H_CTV_ differences.

A rough and intuitive method used to observe the relationship between dose degradation under motion and plan modulation is the comparison of the depth profile of the dose distribution per radiation field. It was observed that patients with a higher MI showed strong dose gradients in the beam path for each individual field. As a consequence, when a large internal motion is detected for these patients; it results in 4DDSim and 4DDReco with target under-dosage or OAR over-dosage in at least some fractions.

## Discussion

This study assessed the plan homogeneity and target volume coverage of 14 patients with locally advanced pancreatic cancer treated with either proton or carbon ion therapy, focusing on intra-fractional motion induced primarily by breathing. It was found that a larger number of treatment sessions deviated from the planned dose distribution, i.e. larger ∆V_95CTV_ (σ_∆v95_) and plan inhomogeneity (σ_∆H_), when the tumor motion amplitude increases (*r* = 0.86 and *r* = 0.77, respectively).

In view of the lack of real-time internal imaging during irradiation, a surrogate signal was used for motion monitoring. The breathing baseline and phase shift, as well as changes in the tumor volume and shape were therefore disregarded in this study.

In terms of motion quantification, the set of patients treated in the prone position showed a mean tumor displacement of (4.8 ± 2.7) mm. Solla et al. [20] have also used the 4DCT but with fiducial markers for motion assessment, which resulted in a larger motion amplitude of (8.5 ± 4.2) mm. This result is again justified by the poor soft tissue contrast of the 4DCT. Tai et al. [21] have measured pancreas motion by relying on 4DCT data only and thus obtained (5.9 ± 2.8) mm, i.e. closest to the one measured for this dataset. On the other hand, where the motion was quantified by Fontana et al. [22] on the basis of MRI data, in which case a better contrast of the pancreas head, body and tail was seen, and patients were secured using immobilization systems (vacuum mattress, mask or abdominal compressor) median values below 2.5 mm were measured.

The quality of the dose distribution using scanned delivery is emphasized as an advantage over passive delivery, as it serves to protect OARs [23]. Having said that, the appearance of interplay can decrease the beneficial impact [24]. Our results showed that six out of fourteen patients showed at least one fraction with V_95CTV_ differences larger than 10%, relative to the static case. On the other hand, the dose heterogeneity increased from an H_CTV_ of (15.9 ± 7.5) % to (27.8 ± 8.5) %. These results might be associated with different factors, such as: (1) patients exhibiting a tumor motion distance larger than 5 mm; (2) dose distribution in the original plan already compromises the target coverage due to the OARs constraints and the V_95CTV_ therefore corresponds to a steeper DVH region; (3) the optimization strategy adopted by the clinical TPS. With respect to the optimization strategy, the plans were evaluated in terms of dose modulation with the aim of correlating this with the dose degradation under motion. Lomax et al. [8] have suggested that IMPT offers potential for delivery with larger range and patient set-up uncertainties compared to the SFUD. This is a consequence of the three-dimensional variation of the beam fluency. Moreover, the TPSs can reach different solutions that might lead to similar dose distributions. This impact would therefore be greater or smaller depending on the optimization strategy and the defined constraints.

Webb et al. [9] have also suggested, in the context of IMRT, that the modulation of a plan should be quantified, in order to understand how the TPS reached the solution, i.e. how the inverse optimization is performed to get the final dose distribution. The application of this concept to this set of patients indicated that patients exhibiting a higher MI and large motion were more susceptible to strong interplay effects. When multiplied by the motion amplitude, the MI was shown to be an indicator of the plan robustness against inter-fractional motion, with a significant linear correlation with the V95_CTV_ and H_CTV_ variation (σ_v95_ and σ_H_) of *r* = 0.76 and *r* = 0.75, respectively.

Nevertheless, the MI presented here cannot be used as a sole indicator of the quality of the delivered dose distribution as this is dependent on other factors including breathing frequency and amplitude, intensity of the raster points with large dose uncertainty and changes in patient anatomy. The MI simply offers additional information enabling us to quantify the probability of dose degradation in view of the interference between the beam and the patient’s breathing. The MI may therefore aide us in selecting between similar dose distributions.

In order to mitigate the impact of the intra-fractional motion, strategies to improve the plan robustness must also be added to the plan optimization process. Robust optimization taking intra-fractional motion into account will automatically lead to less modulation within the fields and will thus result in improved dose coverage [25]. Methods to reduce this impact may also be applied to the treatment delivery (beam gating [26], rescanning [27], or tracking [28]).

We are aware that our study has some limitations. Firstly, our intra-fraction evaluation is based on just a single 4DCT and the internal motion may vary inter-fractionally. In addition, due to the external surrogate signal used, no baseline drifting and amplitude changes of the tumor were taken into account. Sharp et al. [29] have found that phase delays between the internal and external motion and baseline drifting for liver patients with external surrogates would compromise the gated beam delivery. Hence, these aspects must be quantified and considered in future analysis.

In short, for some patients, the intra-fractional motion has the potential to compromise the dose distribution. Particular care should be taken when treating patients with large tumor motion and strategies to reduce its impact must be considered. Beam gating [26] or rescanning [27] are the techniques which offer the greatest potential for use in a clinical routine. More demanding strategies, such as online adjustment of the individual pencil beam energies [28] or 4D-optimised beam tracking [18] are not easily applied using the current beam delivery system and TPS available in our facility.

## Conclusion

The combination of inter-fractional and intra-fractional sources of uncertainties might potentially be used to mitigate the proposed clinical benefit of charged particles when treating pancreatic cancer. Breathing motion monitoring and time-resolved dose calculation might also help in the assessment of robust planning techniques. Therefore, simple strategies such as the selection of beam geometries and the restriction of the plan modulation have been shown to improve the dose delivered to the patient under anatomical change, and might improve the patient outcome.

## Additional file

Additional file 1:Variations per patients of the dose distributions and respective plan modulation evaluation. Table S1 – Mean and standard deviation of the variation of the V_95CTV_ and H_CTV_ for the 4DDSim and 4DDReco and for the respective plans the calculated $\overline{\upsigma \mathrm{np}}$ and MI_plan_. (DOCX 17 kb)

## Abbreviations

4D: time-resolved
4DDRec: 4D Dose Reconstruction
4DDSim: 4D Dose Simulation
BDS: Beam Delivery Sequence
CT: Computed tomography
CTV: Clinical Target Volume
GTV: Gross Tumor Volume
H_CTV_: Homogeneity Dose
IMPT: Intensity-Modulated Particle Therapy
IMRT: Intensity-Modulated Radiation Therapy
ITV: Internal Target Volume
OARs: organs-at-risk
PTV: Planning Target Volume
RBE: Relative Biological Effectiveness
SFUD: Single Field Uniform Dose
TPS: Treatment Planning System
V_95CTV_: CTV receiving at least 95% of the prescribed dose
VFL: Vector Field Length

## References

[1] Siegel RL, Miller KD, Jemal A (2017). Cancer statistics, 2017. CA Cancer J Clin.

[2] Ferlay J, Soerjomataram I, Dikshit R, Eser S, Mathers C, Rebelo M (2015). Cancer incidence and mortality worldwide: sources, methods and major patterns in GLOBOCAN 2012. Int J Cancer Wiley Online Library.

[3] Combs SE, Habermehl D, Kieser M, Dreher C, Werner J, Haselmann R (2013). Phase I study evaluating the treatment of patients with locally advanced pancreatic cancer with carbon ion radiotherapy: the PHOENIX-01 trial. BMC Cancer.

[4] Shinoto M, Yamada S, Terashima K, Yasuda S, Shioyama Y, Honda H (2016). Carbon ion radiation therapy with concurrent gemcitabine for patients with locally advanced pancreatic Cancer. Int J Radiat Oncol Elsevier Inc.

[5] Batista V, Richter D, Combs SE, Jäkel O (2017). Planning strategies for inter-fractional robustness in pancreatic patients treated with scanned carbon therapy. Radiat Oncol.

[6] Bert C, Durante M (2011). Motion in radiotherapy: particle therapy. Phys Med Biol IOP Publishing.

[7] Richter D, Saito N, Chaudhri N, Härtig M, Ellerbrock M, Jäkel O (2014). Four-dimensional patient dose reconstruction for scanned ion beam therapy of moving liver tumors. Int J Radiat Oncol Biol Phys [Internet] Elsevier Inc.

[8] Lomax a J (2014). Intensity modulated proton therapy and its sensitivity to treatment uncertainties 2: the potential effects of inter-fraction and inter-field motions. Phys. Med. Biol [Internet] 2008 cited.

[9] Webb S (2003). Use of a quantitative index of beam modulation to characterize dose conformality: illustration by a comparison of full beamlet IMRT, few-segment IMRT (fsIMRT) and conformal unmodulated radiotherapy. Phys Med Biol Phys Med Biol.

[10] Richter D, Graeff C, Jäkel O, Combs SE, Durante M, Bert C (2014). Residual motion mitigation in scanned carbon ion beam therapy of liver tumors using enlarged pencil beam overlap. Radiother Oncol Elsevier.

[11] Shackleford JA, Kandasamy N, Sharp GC (2010). On developing B-spline registration algorithms for multi-core processors. Phys Med Biol.

[12] Fedorov A, Beichel R, Kalpathy-Cramer J, Finet J, Fillion-Robin J-C, Pujol S (2012). 3D slicer as an image computing platform for the quantitative imaging network. Magn Reson Imaging Elsevier.

[13] Anderle K (2016). In silico trial of photons versus carbon ions in single fraction therapy of lung cancer. Technische Universität Darmstadt.

[14] Lujan AE, Larsen EW, Balter JM, Haken RKT (1999). A method for incorporating organ motion due to breathing into 3D dose calculations. Med Phys.

[15] Steidl P (2011). Gating for scanned ion beam therapy.

[16] Hild S, Durante M, Bert C (2013). Assessment of uncertainties in treatment planning for scanned ion beam therapy of moving tumors. Int J Radiat Oncol Biol Phys.

[17] Richter D (2012). Treatment planning for tumors with residual motion in scanned ion beam therapy. Thesis.

[18] Eley JG, Newhauser WD, Lüchtenborg R, Graeff C, Bert C (2014). 4D optimization of scanned ion beam tracking therapy for moving tumors. Phys Med Biol.

[19] Coghlan A. Little Book of R for Multivariate Analysis [Internet]. Cambridge: Wellcome Trust Sanger Institute; 2017. https://media.readthedocs.org/pdf/little-book-of-r-for-multivariate-analysis/latest/little-book-of-r-for-multivariate-analysis.pdf.

[20] Solla I, Zucca S, Possanzini M, Piras S, Pusceddu C, Porru S (2013). Free ­ breathing conformal irradiation of pancreatic cancer. J. Appl. Clin. Med. Phys..

[21] Tai A, Liang Z, Erickson B, Li XA. Management of respiration-induced motion with 4-dimensional computed tomography (4DCT) for pancreas irradiation. Int. J. Radiat. Oncol. Biol. Phys. [Internet]. Elsevier Inc.; 2013;86:908–913. 10.1016/j.ijrobp.2013.04.012 23688811

[22] Fontana G, Riboldi M, Gianoli C, Chirvase CI, Villa G, Paganelli C (2016). MRI quantification of pancreas motion as a function of patient setup for particle therapy -a preliminary study. J Appl Clin Med Phys.

[23] Shiomi M, Mori S, Shinoto M, Nakayama Y, Kamada T, Yamada S (2016). Comparison of carbon-ion passive and scanning irradiation for pancreatic cancer. Radiother Oncol.

[24] Bert C, Grözinger SO, Rietzel E (2008). Quantification of interplay effects of scanned particle beams and moving targets. Phys Med Biol.

[25] Li H, Zhang X, Park P, Liu W, Chang J, Liao Z, et al. Robust optimization in intensity-modulated proton therapy to account for anatomy changes in lung cancer patients. Radiother Oncol. 2015;114(3):367–7. 10.1016/j.radonc.2015.01.017 PMC4400219 25708992

[26] Mori S, Yanagi T, Hara R, Sharp GC, Asakura H, Kumagai M (2010). Comparison of respiratory-gated and respiratory-ungated planning in scattered carbon ion beam treatment of the pancreas using four-dimensional computed tomography. Int J Radiat Oncol Biol Phys [Internet].

[27] Bernatowicz K, Lomax AJ, Knopf A. Comparative study of layered and volumetric rescanning for different scanning speeds of proton beam in liver patients. Phys. Med. Biol. [Internet]. 2013;58:7905–7920. 10.1088/0031-9155/58/22/7905 24165090

[28] Cheung J (2012). Feasibility of Online Range Adaptive Spot Scanning Proton Therapy. Med. Physics, AAPM Proc.

[29] Sharp GC, Lu HM, Trofimov A, Tang X, Jiang SB, Turcotte J, et al. Assessing residual motion for gated proton-beam radiotherapy. J Radiat Res. 2007;48 10.1269/jrr.48.a55 17513900

