# Supplementary material for: Significance of intra-fractional motion for pancreatic patients treated with charged particles
Source: Radiat Oncol. 2018 Jun 25;13:120. doi: 10.1186/s13014-018-1060-8 (PMC6020245; doi:10.1186/s13014-018-1060-8)
Supplement: Supplementary file 1 — Variations per patients of the dose distributions and respective plan modulation evaluation. Table S1 – Mean and standard deviation of the variation of the V95CTV and HCTV for the 4DDSim and 4DDReco and for the respective plans the calculated $\overline{\upsigma \mathrm{np}}$σnp¯ and MIplan. (DOCX 17 kb) [file 13014_2018_1060_MOESM1_ESM.docx]

Additional file 1

| **Patient** | **∆V95_4DDSim+4DDReco_(%)** | **∆H_4DDSim+4DDReco_(%)** | ${\bar{\boldsymbol{\sigma np}}}_{\boldsymbol{plan}}$ | **MI_plan_** |
| --- | --- | --- | --- | --- |
| **H1** | -8.1±3.0 | 11.7±3.7 | 1.6 | 7.6 |
| **H2** | -2.3±2.2 | 12.7±2.6 | 1.6 | 9.9 |
| **H3** | 1.9±1.4 | 7.8±3.3 | 1.4 | 20.5 |
| **H4** | -7.4±1.9 | 12.7±0.6 | 1.5 | 9.9 |
| **H5** | -5.8±0.6 | 12.1±2.9 | 1.8 | 11.5 |
| **H6** | -10.8±1.5 | 13.2±2.0 | 1.6 | 12.3 |
| **H7** | -20.9±3.9 | 20.7±2.4 | 1.5 | 17.8 |
| **H8** | 2.9±1.8 | 16.3±1.8 | 2.6 | 4.1 |
| **H9** | -3.1±2.2 | 8.1±3.1 | 1.6 | 8.8 |
| **H11** | 1.2±1.0 | 0.6±1.0 | 1.7 | 22.5 |
| **H12** | -11.6±8.6 | 30.0±5.9 | 1.7 | 16.7 |
| **H13** | -15.9±1.9 | 3.2±0.7 | 1.1 | 8.0 |
| **H14** | -3.6±0.7 | 1.3±0.6 | 1.3 | 3.0 |
| **H15** | -6.0±1.5 | 4.2±1.3 | 2.2 | 4.0 |

Table S1 – Mean and standard deviation of the variation of the V_95CTV_ and H_CTV_ for the 4DDSim and 4DDReco and for the respective plans the calculated $\bar{\sigma np}_{plan}$ and MI_plan_ .
